# Klobuchar, NeQuickG, BDGIM, GLONASS, IRI-2016, IRI-2012, IRI-Plas, NeQuick2, and GEMTEC Ionospheric Models: A Comparison in Total Electron Content and Positioning Domains

**DOI:** 10.3390/s23104773

**Published:** 2023-05-15

**Authors:** Yury V. Yasyukevich, Dmitry Zatolokin, Artem Padokhin, Ningbo Wang, Bruno Nava, Zishen Li, Yunbin Yuan, Anna Yasyukevich, Chuanfu Chen, Artem Vesnin

**Affiliations:** 1 Institute of Solar-Terrestrial Physics SB RAS, 664033 Irkutsk, Russia; clausxxx@rambler.ru (D.Z.); annpol@iszf.irk.ru (A.Y.); artem_vesnin@iszf.irk.ru (A.V.); 2 Pushkov Institute of Terrestrial Magnetism, Ionosphere and Radio Wave Propagation, Russian Academy of Sciences, 108840 Moscow, Russia; padokhin@physics.msu.ru; 3 Faculty of Physics, Lomonosov Moscow State University, 119991 Moscow, Russia; chuanfu.chen@physics.msu.ru; 4 Aerospace Information Research Institute (AIR), Chinese Academy of Sciences (CAS), Beijing 100864, China; wangningbo@aoe.ac.cn (N.W.); lizishen@aircas.ac.cn (Z.L.); 5 The Abdus Salam International Centre for Theoretical Physics, I-34151 Trieste, Italy; bnava@ictp.it; 6 Innovation Academy for Precision Measurement Science and Technology (APM), Chinese Academy of Sciences (CAS), Wuhan 430074, China; yybgps@asch.whigg.ac.cn

**Keywords:** ionosphere, models, global navigation satellite systems, Klobuchar, NeQuick, IRI, GLONASS, GEMTEC, positioning, total electron content

## Abstract

Global navigation satellite systems (GNSS) provide a great data source about the ionosphere state. These data can be used for testing ionosphere models. We studied the performance of nine ionospheric models (Klobuchar, NeQuickG, BDGIM, GLONASS, IRI-2016, IRI-2012, IRI-Plas, NeQuick2, and GEMTEC) both in the total electron content (TEC) domain—i.e., how precise the models calculate TEC—and in the positioning error domain—i.e., how the models improve single frequency positioning. The whole data set covers 20 years (2000–2020) from 13 GNSS stations, but the main analysis involves data during 2014–2020 when calculations are available from all the models. We used single-frequency positioning without ionospheric correction and with correction via global ionospheric maps (IGSG) data as expected limits for errors. Improvements against noncorrected solution were as follows: GIM IGSG—22.0%, BDGIM—15.3%, NeQuick2—13.8%, GEMTEC, NeQuickG and IRI-2016—13.3%, Klobuchar—13.2%, IRI-2012—11.6%, IRI-Plas—8.0%, GLONASS—7.3%. TEC bias and mean absolute TEC errors for the models are as follows: GEMTEC—−0.3 and 2.4 TECU, BDGIM—−0.7 and 2.9 TECU, NeQuick2—−1.2 and 3.5 TECU, IRI-2012—−1.5 and 3.2 TECU, NeQuickG—−1.5 and 3.5 TECU, IRI-2016—−1.8 and 3.2 TECU, Klobuchar—1.2 and 4.9 TECU, GLONASS—−1.9 and 4.8 TECU, and IRI-Plas—3.1 and 4.2 TECU. While TEC and positioning domains differ, new-generation operational models (BDGIM and NeQuickG) could overperform or at least be at the same level as classical empirical models.

## 1. Introduction

Mankind involves global navigation satellite systems (GNSS) in different economic activities: autonomous agriculture, transport monitoring, unmanned vehicle transportation, spacecraft navigation, surveying, and much more [1]. Scientists also use GNSS to monitor the state of the environment: sounding the ionosphere [2] and the atmosphere [3], tectonic plate movements [4], natural hazards monitoring [5], etc.

Because most GNSS users still apply single-frequency equipment [6], it is important to compensate for errors caused by the ionosphere. Different methods have been used for this purpose (as recommended by interface control documents of each system). They are first-principles models [7,8], empirical models [9,10], and assimilative models [11,12]. 

The accuracy and precision of the models should be studied to answer which models are better. To evaluate the models’ accuracy, we can use two criteria: how accurate the coordinates are after the models’ corrections (positioning domain) and how accurately the models can estimate the ionospheric total electron content (TEC) (TEC domain). Global ionospheric maps (GIM) [13] provided a huge archive of TEC data, while IGS [14] and other GNSS communities provided a huge archive of GNSS observations.

This current paper compares nine ionosphere models—Klobuchar, NeQuickG, BDGIM, GLONASS, IRI-2016, IRI-2012, IRI-Plas, NeQuick2, GEMTEC—in the TEC domain and the positioning domain based on a data set spanning the period 2014–2020 (entire data set covered 2000–2020, but not all the models can be calculated for the period). The following main scientific questions were addressed: “How accurately do the models calculate TEC?“ and “How precisely can we estimate the receivers’ coordinates using the models to correct for TEC in single-frequency positioning?”

## 2. Materials and Methods

### 2.1. GNSS RINEX Data

**Figure 1:**
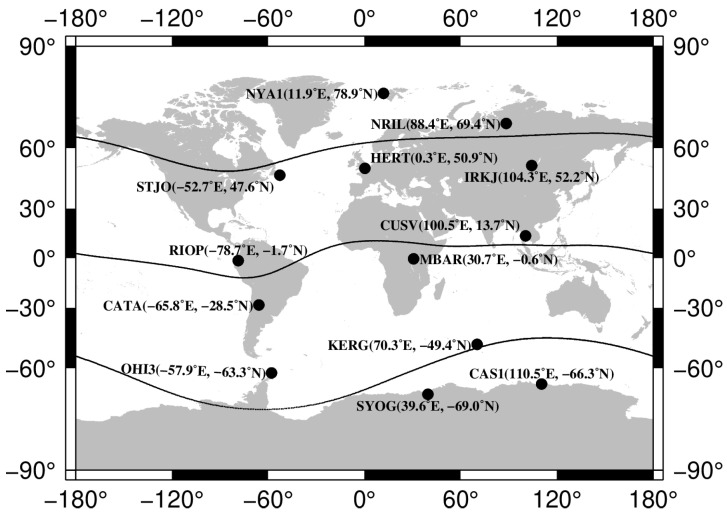
Map of GNSS stations. Labels show station ID and coordinates. Lines show the geomantic equator and 60 deg geomagnetic parallels.

We used GNSS data from 13 IGS stations [14] in different regions. Figure 1 shows the location of the stations. There are two stations at the northern high latitudes and two stations at the southern high latitudes; four stations are located in the equatorial region, and five stations are at mid-latitudes. We tried to choose stations in different longitudinal sectors: 4 are in the American sector, 5 are in the European–African sector, and 5 are in the Asian sector. 

**Figure 2:**
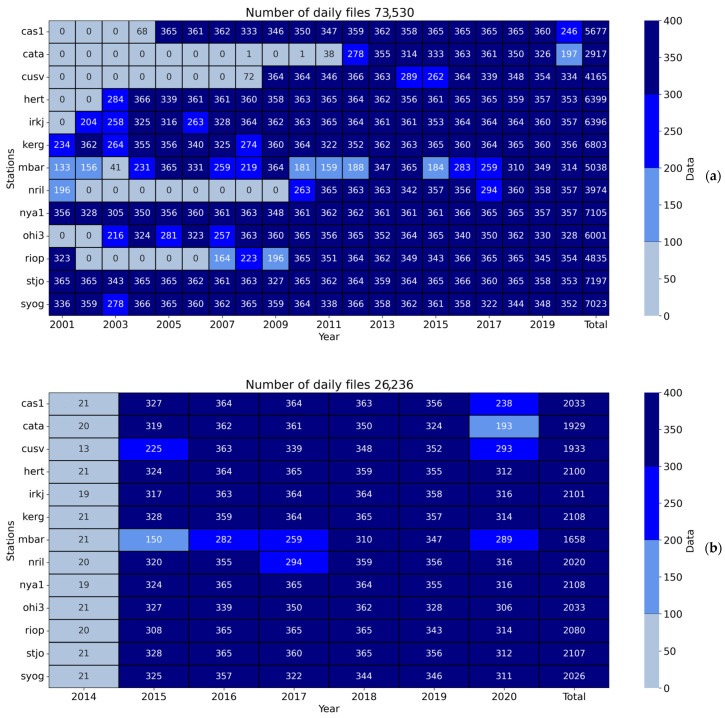
Data sets. (**a**) shows the whole data set, and (**b**) shows the data set when every model allows TEC calculations.

The first data set included data from 2000 to 2020. However, we preferred to exclude the year 2000 to eliminate data when selective availability was turned on. Figure 2 provides detailed statistics for each station for each year. There were from ~2900 to ~7200 daily files for each station. 

Our preliminary analysis showed that our estimations could be biased if we used different periods for different models. It is due to differences in solar and geomagnetic activity and differences in GNSS equipment and signal quality. The bottom panel in Figure 2 shows when data were available for all the models simultaneously. We had 1500–2100 daily files for each station. The data covers the end of 2014 and the entire 2015–2020 because NeQuickG and BDGIM coefficients started to be broadcast after 2014 and 2010, respectively. We used all available data, including those collected during perturbed periods. The analysis of the model’s performance dependence on geomagnetic and solar flux indices is given later in this section.

### 2.2. Ionospheric Models

We used nine ionospheric models: Klobuchar, NeQuickG, BDGIM, GLONASS, IRI-2016, IRI-2012, IRI-Plas, NeQuick2, and GEMTEC. These models require general information such as date–time and location and model-specific inputs such as broadcast coefficients or information about the solar and geomagnetic activity. Table 1 shows the models’ input parameters and some peculiarities.

**Figure 3:**
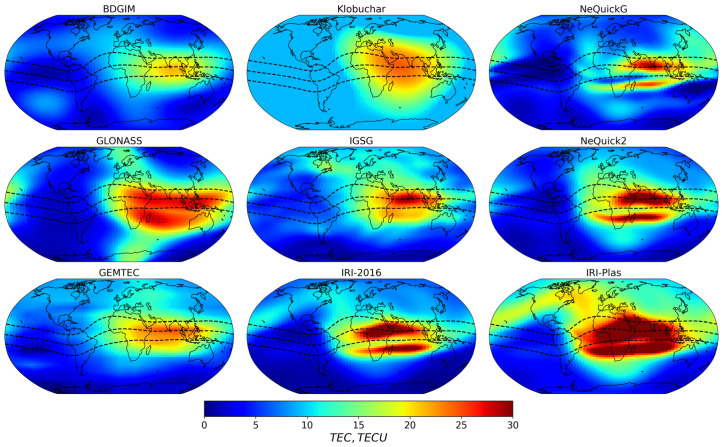
TEC spatial distribution from different models (BDGIM, Klobuchar, NeQucikG, GLONASS, NeQucik2, GEMTEC, IRI-2016, and IRI-Plas) and GIM IGSG data. The model’s name is shown in the subtitle. The data is for 10:00 UT, 1 January 2020.

All the models have different mathematical descriptions and coefficients. Figure 3 shows the spatial TEC distribution at 10:00 UT, 1 January 2020. The models differ in absolute values and time–space TEC peculiarities.

GPS uses *the Klobuchar ionosphere model* for single-frequency correction. The model was developed in the 1970–80s [16]; it has a simple mathematical formulation and, therefore, required low computational costs. Klobuchar model uses constant TEC (9.2 TECU) at nighttime and part of sine function during daytime. Parameters of sine function, which are eight coefficients, are calculated and broadcast through GPS satellite signals. The model provides ~50% ionospheric error correction.

*NeQuick2* [10] is the latest version of ionospheric models based on the sum of Epstein layers [24]. It was developed by the Abdus Salam International Centre for Theoretical Physics (Trieste, Italy) in collaboration with the University of Graz (Austria). The model calculates median dynamics of the ionospheric density profile using 6 semi-Epstein layers with modeled thickness parameters and anchor points defined by foE, foF1, foF2, and M(3000)F2 values, modeled (ITU-R recommendations) or experimentally derived. NeQuick2 uses solar flux (*F10.7*) as input, providing an electron density profile at any location and time. The main advantage of the model is that it is quick-run and has easy calculations along any “ground-to-satellite” ray path.

Using NeQuick formulation, the ESA developed *NeQuickG* [17] for single-frequency ionospheric correction. A Galileo satellite broadcasts three input coefficients (a0, a1, and a2) in the navigation message to compute effective solar flux (Az). NeQuickG uses the effective solar flux as an input to calculate slant TEC, while NeQuick2 uses *F10.7*. NeQuickG also differs from NeQuick2 in the definition of the topside shape parameters. NeQuickG features high performance for single-frequency correction for both ground-based and space-borne users [25,26].

BDS-3 uses a newly designed broadcast ionospheric model *BDGIM*—BeiDou Global Ionospheric delay correction Model—to mitigate the ionospheric delay errors in single-frequency positioning [18]. In contrast to the Klobuchar-like model adopted in the regional BDS-2 system, the BDGIM describes global VTEC distributions with a simplified spherical harmonic expansion referring to a sun-fixed geomagnetic reference frame. The BDGIM is a two-dimensional ionospheric correction model, which relies on an elevation-dependent mapping function to convert ionospheric delays from vertical to slant directions. The nine broadcast parameters drive BDGIM; they are transmitted in a BDS-3 navigation message with an update rate of 2 h. The BDGIM model reduced 25%–98% of the ionospheric error (against GIM IGSG) under different solar activity [19].

The Russian Federal Space Agency suggested a *GLONASS model* for single-frequency ionospheric correction [20]. New GLONASS-K satellites transmit CDMA signals broadcasting three parameters for the adaptive semi-empirical ionosphere model of electron density. We used the TEC formula suggested for ground-based users. The input parameters are *c_A*, *c_F10.7*, *c_Ap*, i.e., the numerical factor for the F2-peak electron density *NmF2*, the corrected value of solar activity index, and daily geomagnetic activity index. We do not have a database of these parameters, so we used *F10.7* (ftp://ftp.seismo.nrcan.gc.ca/spaceweather/solar_flux/daily_flux_values/fluxtable.txt (accessed on 1 January 2022)) and *Ap* (ftp://ftp.gfz-potsdam.de/pub/home/obs/kp-ap/wdc/ (accessed on 1 January 2022)) indexes and *c_A* = 0.95 (an example from [20]).

The international reference ionosphere (IRI) is an international project coordinated and sponsored by the Committee on Space Research (COSPAR) and the International Union of Radio Science (URSI). The IRI models are semi-empirical models of electron density, ion composition, electron and ion temperatures, and other ionospheric parameters. The model ingested data from both ground-based (ionosondes and incoherent scatter radars) and satellite-borne (topside sounders, in situ satellites, and rockets observations). The model input is solar (*F10.7*, *IG*) and geomagnetic (*Ap*) indices, but it can also obtain ionospheric F2 layer critical frequency and maximum ionization height. *IRI-2012* [21] and *IRI-2016* [9] are the two most proven available versions of IRI. In 2022, *IRI-2020* [27] source code appeared at https://irimodel.org/ (accessed on 1 February 2023).

*IRI-Plas* is an IRI model extended with the plasmasphere model [22]. The model calculates electron density until medium Earth GNSS orbits (20,000 km). As input, the model can get the same parameters as IRI and additionally TEC.

The *GEMTEC* model is an empirical global TEC model based on GIM CODG data through a full solar cycle (2000–2009 in the first version [23] and 2000–2012 in the revised one [28]). The model is built on empirical orthogonal functions (on local time, month latitude, solar activity, and the expansion coefficients on longitude) and involves only one parameter—solar activity index *F10.7*.

Klobuchar, NeQuickG, and BDGIM are operational models for GPS, Galileo, and BeiDou systems. Using the broadcast coefficients for the relevant GNSS model, a user can calculate ionosphere correction for positioning. GLONASS model is also an operational model for GLONASS, but for the studied period, the coefficients were not broadcast and are not available now, so it can be considered as a long-term model. *IRI-2016*, *IRI-2012*, IRI-Plas, NeQuick2, and GEMTEC are empirical climatological models. 

### 2.3. Single-Frequency Positioning

We used a GPS-only single-frequency positioning solution and ignored other GNSS. We decided to choose only GPS constellation and signals because GPS has an almost unchanged constellation of about 30 satellites uniformly distributed over the Earth at medium Earth orbits.

To estimate coordinates, we used non-smoothed C/A-code pseudo ranges at GPS L1 frequency. The elevation cut-off was 15°. Troposphere delay correction used the Saastamoinen model [29]. To estimate coordinates, we used a typical iterative solution [1].

We developed software, NAVI [30], that processes RINEX data and calculates geocentric coordinates *X*, *Y*, and *Z* for single-frequency positioning. In this article, NAVI software used broadcast ephemeris to show a real condition for most users. We had 30 s estimates of coordinates and corresponding positioning errors.

### 2.4. Total Electron Content from Global Ionosphere Maps

Global ionospheric maps provide a huge amount of data [13]. We used IGSG combined product. GIM IGSG includes TEC data with 2 h time resolution and 2.5°/5° resolutions in latitude/longitude. An example of GIM IGSG is shown in Figure 3 (middle panel).

IGSG maps cover the whole period from 1998 to date. IGSG TEC was compared with model results, and it was also implemented for single-frequency correction to compare with correction quality for other models.

### 2.5. Approach for Estimations

To evaluate model performance, we used two approaches: the positioning domain and the TEC domain.

#### 2.5.1. Positioning Domain

We applied different models’ corrections for pseudo-range observables and then compared 3D positioning errors for these solutions.

As a true position, we used precise coordinates obtained through SOPAC SCOUT (http://sopac-old.ucsd.edu/scout.shtml accessed on 11 April 2023). To check the SOPAC SCOUT results we compared it with our median values of *X*, *Y*, *Z*. As positioning error, we consider the three-dimensional error—root-mean-square deviation from the true position *X*_0_, *Y*_0_, and *Z*_0_ as follows:

(1) $$\sigma =\sqrt{\Delta X^{2}+\Delta Y^{2}+\Delta Z^{2}},$$

where Δ*X* = *X* − *X*_0_, Δ*Y* = *Y* − *Y*_0_, Δ*Z* = *Z* − *Z*_0_. 

To avoid issues due to ephemeris errors, we excluded data when the positioning error exceeds 50 m.

The better the model in the positioning domain, the smaller error *σ*.

#### 2.5.2. TEC Domain

There are several approaches to validate models in TEC domains. The first one is to compare experimental and model variations in slant TEC for an individual satellite-to-receiver line of sight [31]. Another one is to compare absolute vertical TEC from the model with well-validated reference, for example, IGS final Global Ionospheric Map. Both approaches have advantages and drawbacks. In our case, we directly compared absolute vertical TEC from model *I^M^* with TEC from Global ionosphere maps IGSG *I^GIM^* (for *N* measurements). Four parameters were used as error proxies.

- Mean TEC error (TEC bias) <Δ*I*>,

(2) $$\langle \Delta I\rangle =\frac{1}{N}\sum_{i=1}^{N}\left(I_{i}^{M}-I_{i}^{GIM}\right);$$

- Mean absolute TEC error (MAE) <|Δ*I*|>,

(3) $$\langle \left|\Delta I\right|\rangle =\frac{1}{N}\sum_{i=1}^{N}\left|I_{i}^{M}-I_{i}^{GIM}\right|;$$

- Mean percentage TEC error (MPE) <Δ*I*/*I*>,

(4) $$\langle \Delta I/I\rangle =100\%\cdot \frac{1}{N}\sum_{i=1}^{N}\frac{\left(I_{i}^{M}-I_{i}^{GIM}\right)}{I_{i}^{GIM}};$$

- Mean absolute percentage TEC error (MAPE) <|Δ*I*|/*I*>,

(5) $$\langle \left|\Delta I\right|/I\rangle =100\%\cdot \frac{1}{N}\sum_{i=1}^{N}\frac{\left|I_{i}^{M}-I_{i}^{GIM}\right|}{I_{i}^{GIM}}.$$

The better the model in the TEC domain, the smaller absolute values of errors (2–5).

In the literature, different statistical parameters have been used as performance indicators (e.g., RMSE or MAE [32]) In this work, MAE has been chosen as a reference, also considering that in general, all the other parameters exhibited a similar behavior.

## 3. Ionospheric Model Quality

### 3.1. Ionospheric Models in Positioning Domain

**Figure 4:**
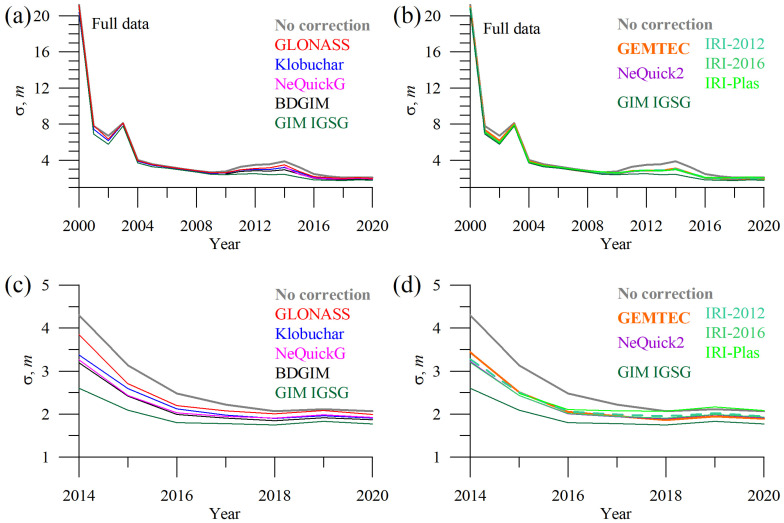
Dynamics of the yearly mean three-dimensional single-frequency positioning error with different ionospheric corrections: GLONASS (red), Klobuchar (blue), NeQuickG (magenta), BDGIM (black), GIM IGSG (forest green), GEMTEC (orange), NeQuick2 (purple), IRI-2012 (sea green), IRI-2016 (light green), and IRI-Plas (green). (**a**,**c**) show the data for operational models; (**b**,**d**) show the data for empirical models; all the panels show errors of GIM-based positioning and positioning without ionospheric correction.

The dynamics of 3D-error changes in the solar cycle. The year 2000 features the greatest error, as seen from Figure 4, where the errors peak. Until May 2000, selective availability was on, so we excluded the year 2000 from our analysis. 

Positioning error depends on the solar cycle phase, and, in general, they decrease as solar activity decreases. Applying GIM data, the errors differ in 2008 and 2018. To avoid bias caused by different statistics, we used data during 2014–2020 only when all the models were executable below.

From Figure 4, we see that all models perform in a similar way, and, in particular, IRI and NeQuick are very close to each other, while the Klobuchar and GLONASS models provide slightly worse corrections. All the models improve the position estimates with respect to the case when no ionospheric correction is applied but cannot achieve the performance of the GIM-corrected solutions.

**Figure 5:**
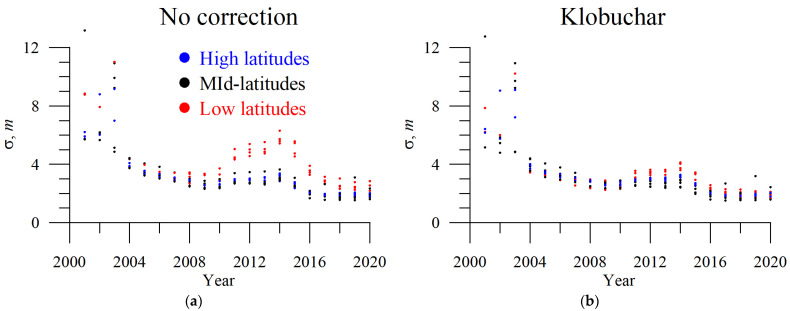
Dynamics of the yearly mean single-frequency positioning error at different stations at high latitudes (blue dots), mid-latitudes (black dots), and low latitudes (red dots). (**a**) shows the dynamics without ionospheric correction, and (**b**) shows the dynamics with Klobuchar ionospheric correction.

Each station features its own error, so mean values are smoothed. Figure 5 shows yearly mean 3D positioning error dynamics without ionospheric correction (a) and with the Klobuchar-based correction (b) at each station at high (blue dots), mid- (black dots), and low (red dots) latitudes. Low-latitude stations (CATA, CUSV, RIOP, and MBAR in red dots) depend stronger (compared with mid- and high latitude stations) on the solar cycle. We see that correction is most effective for low-latitude stations, where high TEC values in the equatorial anomaly crest produce high errors in non-corrected single-frequency GNSS positioning.

Table 2 reports the mean 3D error and the previously mentioned statistical parameters (Section 2.5.2) for different models. The models were sorted by σ3D from smallest to highest. The differences in mean σ3D for models are small. However, due to the huge data set (~75 million independent measurements), the differences are statistically significant at the level (at least) α < 0.001 except GEMTEC vs. IRI-2016 difference. 

**Figure 6:**
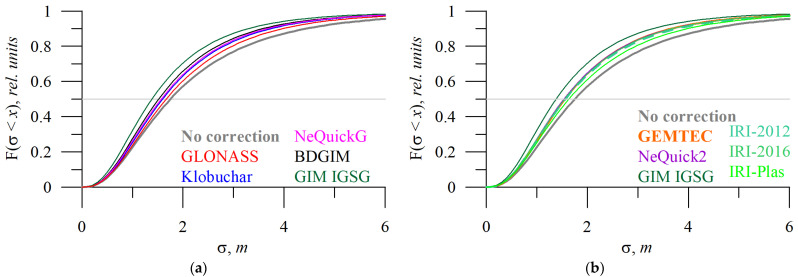
Statistics of positioning errors. The cumulative distribution function of 3D error of single-frequency positioning obtained without (**a**,**b**, grey) and with ionospheric correction by different models: GLONASS (**a**, red), Klobuchar (**a**, blue), NeQuickG (**a**, magenta), BDGIM (**a**, black), GIM IGSG (**a**,**b**, forest green), GEMTEC (**b**, orange), NeQuick2 (**b**, purple), IRI-2012 (**b**, sea green), IRI-2016 (**b**, light green), and IRI-Plas (**b**, green). The horizontal light grey line shows a 0.5 level.

Figure 6 shows the Cumulative Distribution Function of the observed 3D errors—i.e., the fraction of cases (with respect to the total) having an error less than or equal to a given value. The closer the distribution line is to the *y*-axis, the better the model performance is. The difference in operational models can be clearly seen: BDGIM features the best performance, while GLONASS features the worst one. Empirical models feature close distributions, NeQuick2 being slightly better and IRI-Plas a little worse.

**Figure 7:**
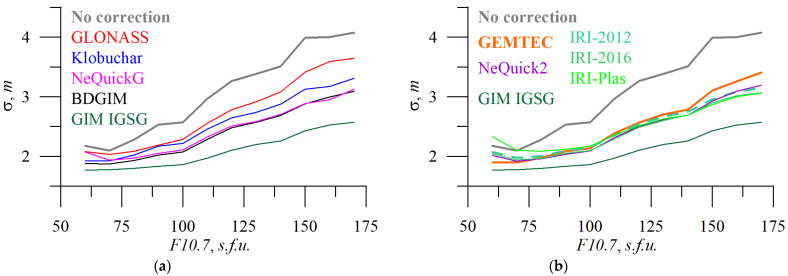
Solar activity influences positioning errors. *F10.7* dependence of 3D error of single-frequency positioning without applying (**a**,**b**, grey) and applying ionospheric corrections from different models: GLONASS (**a**, red), Klobuchar (**a**, blue), NeQuickG (**a**, magenta), BDGIM (**a**, black), GIM IGSG (**a**,**b**, forest green), GEMTEC (**b**, orange), NeQuick2 (**b**, purple), IRI-2012 (**b**, sea green), IRI-2016 (**b**, light green), and IRI-Plas (**b**, green).

**Figure 8:**
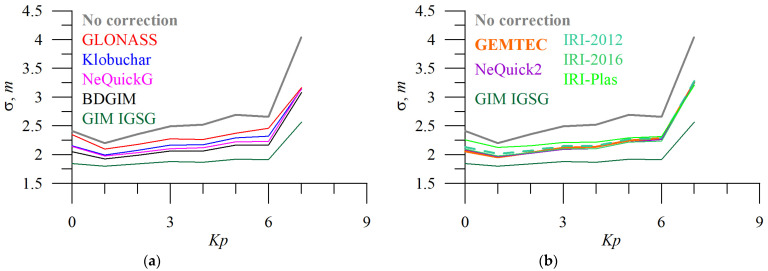
Geomagnetic activity influence on positioning error. *Kp* dependence of 3D error of single-frequency positioning without applying (**a**,**b**, grey) and applying ionospheric corrections from different models: GLONASS (**a**, red), Klobuchar (**a**, blue), NeQuickG (**a**, magenta), BDGIM (**a**, black), GIM IGSG (**a**,**b**, forest green), GEMTEC (**b**, orange), NeQuick2 (**b**, purple), IRI-2012 (**b**, sea green), IRI-2016 (**b**, light green), and IRI-Plas (**b**, green).

Figure 7 and Figure 8 show how *F10.7* and *Kp* influence the 3D positioning errors for different models. Linear dependence for *F10.7* dependence is quite clear. The slope shows the difference in correction effectiveness. We should note the relatively low efficiency of GEMTEC. Among operational models, GLONASS features the worst results (probably due to the incorrectly used “correction factor”).

All the models feature high errors during magnetic storms. It is not only due to unexpected TEC dynamics but also due to irregularities influence that deteriorates positioning. Up to *Kp* = 6, geomagnetic activities faintly influences single-frequency positioning.

### 3.2. Ionospheric Models in TEC Domain

Table 2 summarizes also errors in the TEC domain for all the models for the period when we have all the models together (2015–2020 is the declining phase and solar minima). IRI-Plas and Klobuchar overestimate TEC, while other models underestimate TEC. 

Errors in the positioning domain differ from those in the TEC domain. With reference to mean absolute TEC error, GLONASS and Klobuchar show the highest errors in the TEC domain, while empirical IRI-2012, IRI-2016, and NeQuick2 models show similar results. GEMTEC and BDGIM models show the best results.

GEMTEC model features the smallest TEC bias. This is because GEMTEC is based on CODG GIM, but input data for the model covered only 2000–2009, while, here, we used another solar cycle for testing (2015–2020). Additionally, BDGIM relies on GIM, so it displays a small TEC bias and mean absolute error.

**Figure 9:**
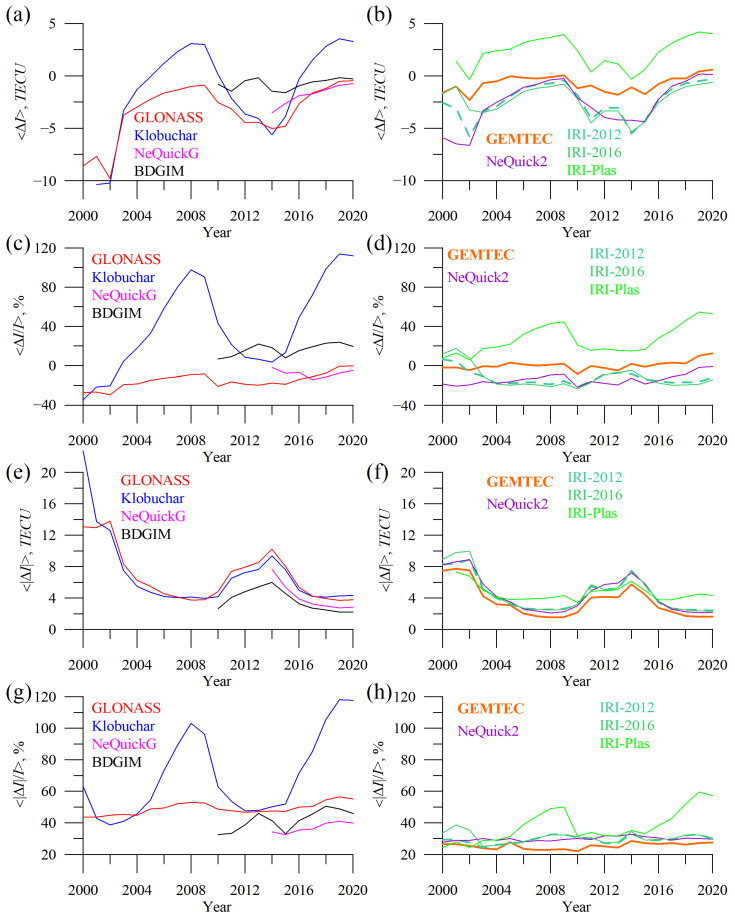
Dynamics of the yearly mean TEC error: GLONASS (red), Klobuchar (blue), NeQuickG (magenta), BDGIM (black), GEMTEC (orange), NeQuick2 (purple), IRI-2012 (sea green), IRI-2016 (light green), and IRI-Plas (green). (**a**,**b**) show TEC bias, (**c**,**d**) show mean absolute TEC error, (**e**,**f**) show mean percentage TEC error, (**g**,**h**) show mean absolute percentage TEC error.

Figure 9 shows the dynamics of TEC error for different models. The errors vary as a function of solar activity. The highest absolute errors occur at solar maxima, while the highest relative errors (especially for the Klobuchar model) occur at solar minima.

TEC biases (Figure 9a,b) vary mostly within ±5 TECU. All the models except GEMTEC and BGDIM show a drop in TEC bias during solar maximum. IRI-Plas overestimate TEC except during solar maxima. BDGIM slightly underestimates TEC during all periods, but the TEC bias is stable. The Klobuchar model displays a high error and significant change in mean TEC bias.

The mean relative TEC error reaches 100% for Klobuchar and is at ~−20% for GLONASS, NeQuick2, IRI-2012, and IRI-2016. Huge variations in Klobuchar’s mean relative TEC error are due to its constant values at night. To better visualize this feature, we considered local time dependence for different models.

Except for BDGIM, absolute TEC errors (Figure 9e,f) of the operational model exceed those of the empirical ones. 

GLONASS model exhibits the highest mean absolute error, while BDGIM—is the lowest. NeQuickG features lower MAPE than BGIM but higher MAE. The mean absolute TEC error in 2014 (when we have data for all the models but a poorer statistic for NeQuickG) reached 7.7–12.7 TECU. 

There are large differences in the statistical parameters that we used for precision/accuracy estimation. Even in the TEC domain, a model could be better in one parameter but worse in the other. The MAPE of BDGIM exceeds that of NeQuickG; it is the opposite of the mean absolute error. That is due to where the highest errors occur. 

The mean absolute percentage error (Figure 9g,h) for NeQucik2 is stable during two solar cycles. Such stability is typical for GLONASS, IRI-2012, IRI-2016, GEMTEC, and probably for NeQucikG (but we do not have enough data to check the latter).

**Figure 10:**
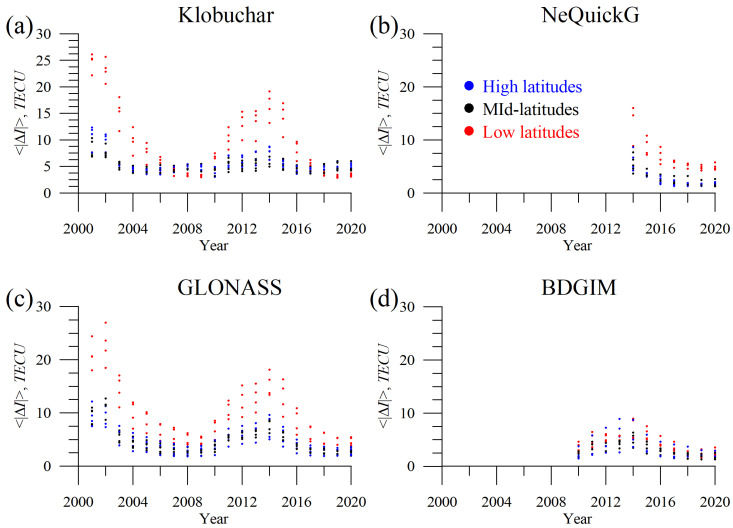
Dynamics of the yearly mean absolute TEC error at different stations (Figure 1) at high latitudes (blue dots), mid-latitudes (black dots), and low latitudes (red dots) for Klobuchar (**a**), NeQuickG (**b**), GLONASS (**c**), and BDGIM (**d**) models.

While Figure 9 considers yearly mean global parameters, Figure 10 shows the absolute TEC error for single GNSS stations in the same way as Figure 5. 

For all models, the highest errors are obtained for solar maxima and the lowest for solar minima. Equatorial stations feature higher errors for all the models except BDGIM. The latter features similar errors at high, mid-, and low latitudes.

**Figure 11:**
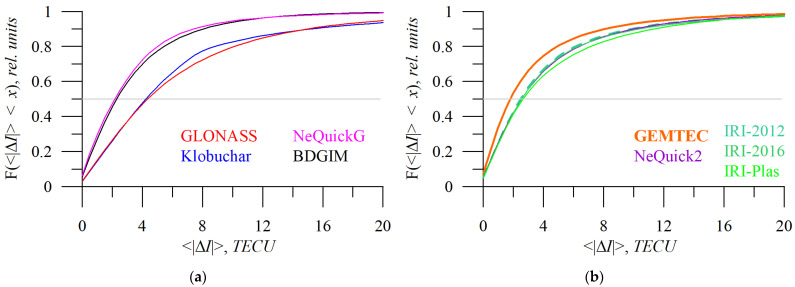
The cumulative distribution function of absolute TEC error by different models: GLONASS (**a**, red), Klobuchar (**a**, blue), NeQuickG (**a**, magenta), BDGIM (**a**, black), GEMTEC (**b**, orange), NeQuick2 (**b**, purple), IRI-2012 (**b**, sea green), IRI-2016 (**b**, light green), and IRI-Plas (**b**, green). The horizontal light grey line shows a 0.5 level.

**Figure 12:**
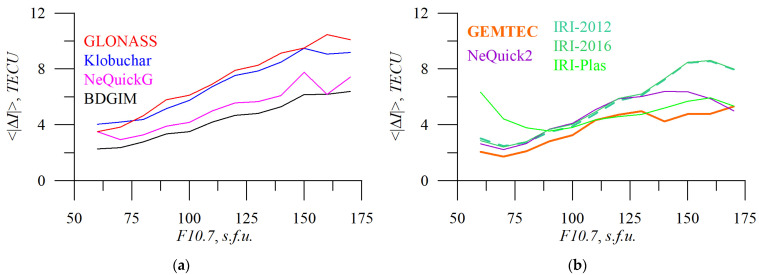
Solar activity influence on absolute TEC errors. *F10.7* dependence of absolute TEC errors for the following different models: GLONASS (**a**, red), Klobuchar (**a**, blue), NeQuickG (**a**, magenta), BDGIM (**a**, black), GEMTEC (**b**, orange), NeQuick2 (**b**, purple), IRI-2012 (**b**, sea green), IRI-2016 (**b**, light green), and IRI-Plas (**b**, green).

**Figure 13:**
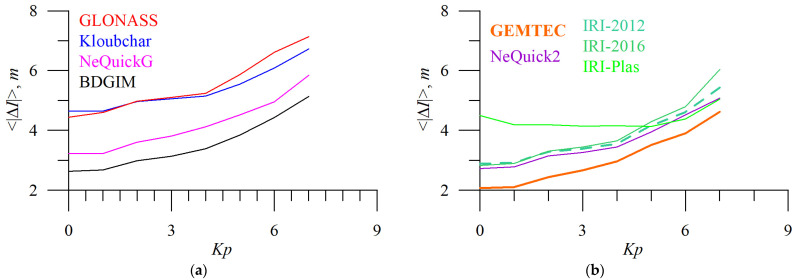
Geomagnetic activity influence on absolute TEC errors. *Kp* dependence of absolute TEC errors for the following different models: GLONASS (**a**, red), Klobuchar (**a**, blue), NeQuickG (**a**, magenta), BDGIM (**a**, black), GEMTEC (**b**, orange), NeQuick2 (**b**, purple), IRI-2012 (**b**, sea green), IRI-2016 (**b**, light green), and IRI-Plas (**b**, green).

**Figure 14:**
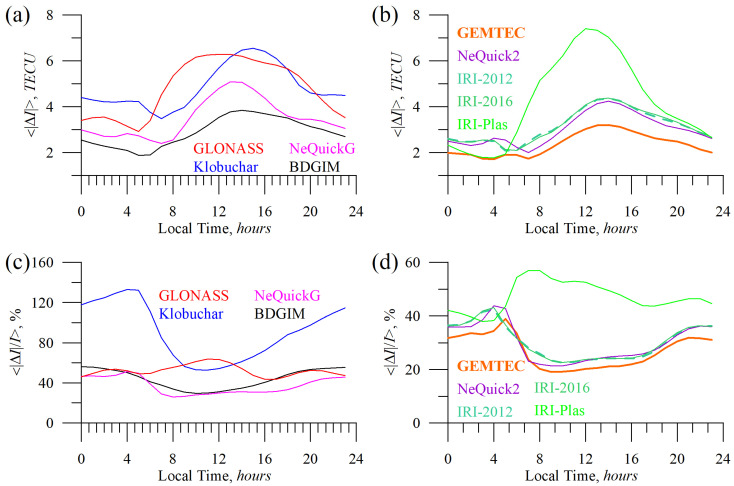
Absolute TEC errors vs. local time: GLONASS (red), Klobuchar (blue), NeQuickG (magenta), BDGIM (black), GEMTEC (orange), NeQuick2 (purple), IRI-2012 (sea green), IRI-2016 (light green), and IRI-Plas (green). (**a**,**b**) show mean absolute TEC error, (**c**,**d**) show mean absolute percentage TEC error.

**Figure 15:**
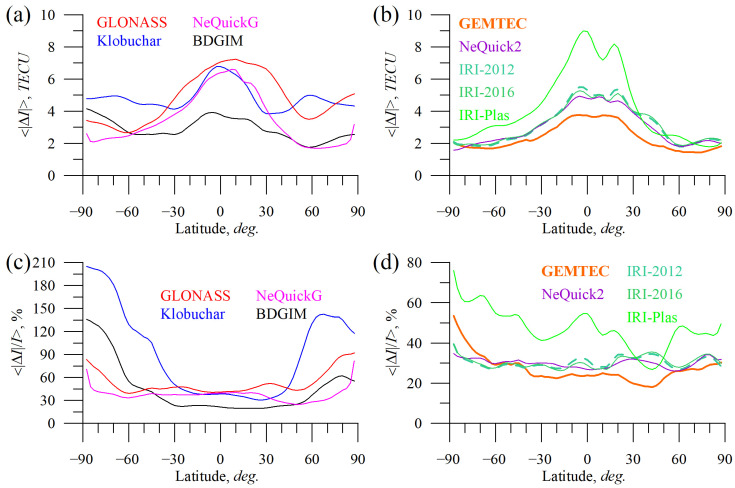
Absolute TEC errors vs. latitude: GLONASS (red), Klobuchar (blue), NeQuickG (magenta), BDGIM (black), GEMTEC (orange), NeQuick2 (purple), IRI-2012 (sea green), IRI-2016 (light green), and IRI-Plas (green). (**a**,**b**) show mean absolute TEC error, (**c**,**d**) show mean absolute percentage TEC error.

**Figure 16:**
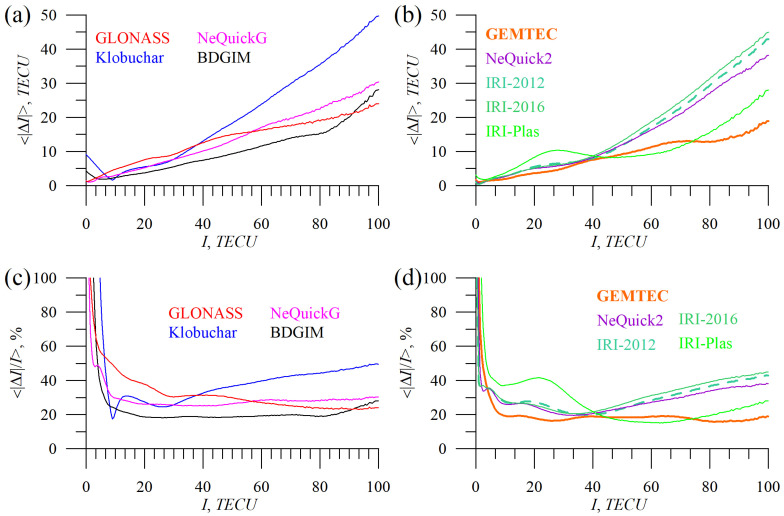
Absolute TEC errors vs. total electron content from GIM IGSG: GLONASS (red), Klobuchar (blue), NeQuickG (magenta), BDGIM (black), GEMTEC (orange), NeQuick2 (purple), IRI-2012 (sea green), IRI-2016 (light green), and IRI-Plas (green). (**a**,**b**) show mean absolute TEC error, (**c**,**d**) show mean absolute percentage TEC error.

The cumulative distribution functions of absolute TEC error (Figure 11) show a remarkable difference between Klobuchar/GLONASS and NeQuickG/BDGIM models. The data for Figure 11, Figure 12, Figure 13, Figure 14, Figure 15 and Figure 16 cover periods when all the models appeared in the data set (2015–2020). 

From the cumulative distributions (panel a), we see that for small errors (~5 TECU) NeQuickG performs slightly better than BDGIM, while for errors greater than ~10 TECU, the distributions are similar. This reflects the peculiarities pointed out in Figure 9, where the mean absolute error for NeQuickG exceeds those for BDGIM. Table 3 summarizes 25%, 50%, 75%, 90%, and 95% error percentiles (*Y*), i.e., what amount of data (*Y*) has an error less than the one shown in the table.

In Table 3, the models are ranked in accordance to positioning domain error (σ_3D_), from the least to the most accurate. If instead, we consider the TEC domain, e.g., 90% and 25%, we could rank the models as GEMTEC, NeQuickG, BDGIM, NeQucik2, IRI-2012, IRI-2016, IRI-Plas, Klobuchar, and GLONASS.

Figure 12 and Figure 13 show the dependence of absolute TEC error on solar (Figure 12) and geomagnetic (Figure 13) activity.

The operational models’ TEC errors depend on *F10.7* almost linearly, while empirical models feature stronger variations. IRI-Plas shows higher errors at low *F10.7*. Obtained TEC error dynamics can be used to improve ionospheric models.

We should note that under typical conditions (*F10.7* within 60–160 s.f.u. or *Kp* within 0–7) BDGIM performs better than the NeQuickG model. This disagrees with the previous ranking from the cumulative distribution of the error.

To understand the discrepancies in our estimation for different models, Figure 14, Figure 15 and Figure 16 have been produced. They show mean absolute and mean absolute percentage models’ errors vs. local time (Figure 14), geographic latitude (Figure 15), and total electron content (Figure 16).

From Figure 14, it is evident that models’ errors depend on local time. The IRI-Plas model, taking into account the plasmasphere, shows the highest errors during daytime with a maximum of 12 LT. Previously, we mentioned that the model overestimated IGSG TEC. However, MAPE is maximum at sunrise when TEC is small.

The Klobuchar model’s absolute TEC error is maximum at 15 LT, while MAPE is maximum at night when the Klobuchar model provides constant TEC. GLONASS error also peaks during the daytime.

The mean percentage error of NeQuickG and BDGIM is lower if compared with GLONASS and Klobuchar. BDGIM features the weakest LT dependence. NeQuickG, NeQucick2, and IRI-2012/IRI-2016 models peak in absolute error at ~14 LT, while the highest relative error is found at night.

The latitudinal dependence of TEC errors (Figure 15) reveals that almost all the models’ maximum error occurred at low latitudes, except for BDGIM. It is interesting to note that the Klobuchar model features a local maximum at 60°N, while GLONASS shows a local minimum. Probably, it is due to data coverage involved in the model design. It was unexpected that BDGIM shows the highest mean absolute error at high latitudes, where its precision almost reaches the Klobuchar model level (in the Southern hemisphere). GEMTEC features the lowest errors among the models; the errors peak at equatorial latitudes.

Figure 16 demonstrates how the models’ errors vary with TEC magnitude. All the models increase in error when TEC increases but with different trends. We noted a sharp increase in the Klobuchar model error at high TEC values. The model is also insufficient to reproduce small TEC values, which is why it features high MAPE at high latitudes (Figure 15).

We see a sharp increase in BDGIM error at high TEC (which can be an additional point to improve the model). The main difference between IRI-2012 and IRI-2016 appears at high TEC values. We should note, that if at low TEC IRI-Plas, errors exceed (up to two times) those of IRI-2012/IRI-2016/NeQuck2 models, then at high TEC values, IRI-Plas performance is better.

## 4. Discussion

We studied the performance of nine ionospheric models in TEC and positioning domains during 2014–2020 (the full data set covered 2000–2020).

The main result is that all models perform in a similar way as far as the position domain is concerned (this could be due to noise and other factors (e.g., broadcast ephemeris). The improvement in positioning error against noncorrected solutions was GIM IGSG at 22.0%, BDGIM at 15.3%, NeQuick2 at 13.8%, GEMTEC at 13.3%, NeQuickG and IRI-2016 at 13.3%, Klobuchar at 13.2%, IRI-2012 at 11.6%, IRI-Plas at 8.0%, and GLONASS at 7.3%. While the difference is not high, it is statistically significant. We should also note that instantaneous results should show higher RMS than median ones [33]. So, some reasons for the higher RMS in our research is that we used instantaneous coordinates and TEC values.

We noticed that the models have a different ranking whether the TEC domain or positioning domain is considered. Yet, the GEMTEC model exhibits the smallest global mean absolute error <|Δ*I*|> and the least TEC bias. TEC bias and mean absolute TEC errors are as follows: −0.3 and 2.4 TECU for GEMTEC; −0.7 and 2.9 TECU for BDGIM; −1.2 and 3.0 TECU for NeQuick2; −1.5 and 3.2 TECU for IRI-2012; −1.5 and 3.5 TECU for NeQuickG; −1.8 and 3.2 TECU for IRI-2016; 1.2 and 4.9 TECU for Klobuchar; −1.9 and 4.8 TECU for GLONASS; 3.1 and 4.2 TECU for IRI-Plas. 

Different scientists used the positioning domain along with the TEC domain to assess the model performance [28,34]. Rovira-Garcia et al. [34] clearly formulated the advantage of such an approach. Using data for the year 2014 and 34 permanent stations, they calculated both residual dual-frequency slant TEC (that is carrier-to-phase leveled) vs. GIM TEC and positioning error in PPP single-frequency solution involving GIM IGSG and GIM UQRG. The measurement noise, pseudo-range multipath, evaluation metric, and outliers are the main problems in the models’ evaluation, and if one takes them into account, then positioning and TEC domain are close [34,35]. The technique allowed the authors to reveal that GIM UQRG is better than GIM IGSG both in TEC and positioning domains. We did not perform carrier-to-phase leveling and used a standard non-smoothed coordinate solution with a broadcast ephemeris because this reveals the real problems in standard positioning that appear for most users. We compensated for this decrease in sensitivity with a huge data amount (6 years to compare all the models together and 20 years for older models).

Usually, the models were tested for a rather limited time period and region covering. Ivanov et al. [28] used only the 22nd day of each month from 2001 to 2011 to test the Klobuchar and GEMTEC models and found that they eliminate about 57 and 85% of the absolute error. Table 2 and Table 3 also demonstrate that the GEMTEC model performs better than the Klobuchar model. We should note that in the TEC domain, GEMTEC is one of the best models.

Ivanov et al. [36] showed that GEMTEC overperformed the Klobuchar model for 2003–2012 (at the Asian mid-latitudes). Because GEMTEC is a GIM-based model involving data up to 2012, we could expect a reduction in its precision after that period. Zhukov et al. [37] showed that GEMTEC overperformed NeQuick2 and especially Klobuchar models for 2017. Current results show that GEMTEC keep good quality after 2012 and overperformed IRI and NeQuick sets after 2012 until 2020 at almost the same level as before.

Several studies found that the IRI-Plas model overestimates TEC [38]. Okoh et al. [39] comprehensively studied the NeQuick2 and IRI-Plas over the whole solar cycle (2006–2017) based on 36 globally distributed stations (we used three of those stations in our set). The NeQuick2 overperformed IRI-Plas in the TEC domain except for several locations (three stations in the African–Asian sector). IRI-Plas overestimates vTEC, while NeQuick2 underestimates vTEC during high solar activity and overestimates it during local daytime for low and moderate solar activity (but not as much as IRI-Plas). Our results confirm that IRI-Plas (and Klobuchar model) overestimate TEC, while other studied models underestimate it.

Several studies showed that IRI-Plas overestimates electron density in the topside ionosphere [40,41]. The IRI-Plas significantly overestimate vertical TEC, especially during night-time and winter periods. IRI-2021 reproduced vertical TEC better [40]. Our results agree that IRI-Plas in general overestimate vertical TEC but disagree for the local time dependence; we observed the highest IRI-Plas errors during daytime (both relative and absolute). We should note that the IRI-Plas models were updated during this time and that actually, we used solar cycle 25 data, while Zakharenkova et al. [40] considered solar cycle 24. We should note that IRI-2012 and IRI-2016 overperform IRI-Plas (with the parameters we choose) in both TEC and positioning domains. Gordienko et al. [42] confirm our results and show that IRI-Plas-TEC values are significantly larger than those of daytime GIM-TEC at all locations of Russia and Kazakhstan and for all levels of solar activity. Nevertheless, IRI-Plas could underestimate 30% of TEC during the nighttime equinox [41]. Our results show that at least during nighttime and early morning hours, IRI-Plas performs almost the same way as other climatological empirical models.

Maltseva et al. [43] studied the Neustrelitz Global Model (similarly to GEMTEC the model is based on GIM CODG) and IRI-Plas; they mentioned that the IRI-Plas featured higher errors at low latitudes. We observed an increase in TEC error for all the models at the low latitudes, but the biggest errors are for the IRI-Plas model in the low-latitude region. Hoque et al. [44] suggested modifying Neustrelitz Model to use the Galileo broadcast model. Such a model also overperformed the Galileo operational NeQuickG model [44] and is comparable with NeQuickG without Galileo broadcast coefficients [45]. It agrees with our results for a similar model GEMTEC, but deep checking Neustrelitz Model should be studied further. 

Li et al. [46] studied the performance of the IRI-2012 model over Chinese mid-latitudes and found that the model underestimated nighttime TEC during both solar minima and solar maxima; it had difficulties in capturing the solar activity component of TEC and it underestimated annual and one-third annual periodic amplitude. We also confirm that when *F10.7* increases the IRI-2012 and IRI-2016, models exhibit an increase in TEC errors larger than NeQuick2, GEMTEC, or IRI-Plas.

The IRI-2012 model agrees with GPS-TEC at equatorial latitudes during nighttime quiet periods, but during the daytime, it underestimates GPS-TEC [47]. This fact could be related to an increase in the observed TEC error during the daytime (Figure 14). 

The performance of IRI models (at least for IRI-2001 and IRI-2007) decreases almost linearly when *F10.7* increases and nonlinearly when *Kp* increases [48]. Here, we showed the same *Kp*-pattern for all the models, but we did not analyze periods with high *Kp*. We should note that there is no significant error dependence on *Kp* for the IRI-Plas model. While the TEC error increases linearly with *F10.7* for all the models, for IRI-Plas, at low *F10.7*, we note higher errors. In the positioning domain, we observe a sharp increase in σ3D even during slightly disturbed geomagnetic periods. That can be due to ionospheric irregularity that starts to play an important role [49]. In their pioneering paper, Ephishov et al. [50] found that IRI models (IRI-90 and IRI-95) overestimated TEC at midlatitudes; however, Kenpankho et al. [51] showed that it is different at low latitudes where the IRI-2007 underestimated TEC. We should note that the IRI topside was improved significantly in the new-generation models [9]. 

Angrisano et al. [52] showed that NeQuick overperforms the Klobuchar model in 98% of TEC estimates. However, in the positioning domain, the improvement is not so high—only 4–5%. In this current research, small differences in the positioning domain are, therefore, not surprising. However, they are statistically significant at α < 0.001 due to the huge data set.

Wang et al. [53] validated NeQuick2 during 2008–2021. They found an increase in TEC RMSE under solar maxima and mean TEC bias at ~−1.5 TECU. That agrees with our results of TEC bias at ~−1.22 and overall mean absolute TEC error increase in both solar maxima. 

Orus Perez [54] showed that NeQuickG yields lower RMS of the precise point positioning error than the Klobuchar model: 20% and 11% for the horizontal and vertical components, respectively. The GIM significantly overperformed both models: 37 and 27% in the horizontal and vertical navigation error components, respectively. In the precise point positioning domain [54], NeQuickG RMS of positioning error is 50 cm less than of the Klobuchar model. Our results did not show such a big difference in the positioning domain between NeQuickG and Klobuchar models but did show it in the TEC domain. The probable reason is the noises due to non-smoothed pseudo ranges. Single-frequency PPP showed that GIM provides ~1.5–2.5 better positioning than IRI-2016 and NeQuick [55].

In general, IRI-2016 and NeQuick2 showed similar statistical results [55]. However, our results show that NeQuick2 has a smaller TEC bias than IRI-2016.

Wang et al. [19] estimated BDGIM coefficients (which should be broadcast) for 2010–2017 (BeiDou broadcasts coefficients from 2015). They found that BDGIM performs similarly to the NeQuickG model and about 5% better than the empirical IRI-2016 model. Our results for the TEC domain showed that BDGIM overperformed other operational models (up to 20%) and had significantly less TEC bias.

We should note that in our research, the GLONASS model was not used as an operational model because the broadcast model coefficients were not available. We used *F10.7* and *Ap* indexes instead for their effective analogues, which are implied for the model. So, the quite poor results obtained are most probably due to a non-optimal use of driving parameters.

Our results surprisingly revealed that the empirical median NeQuick2 model tends to perform slightly better than NeQuickG (which implied corrections based on actual data), both in positioning and TEC domain. This demonstrates the importance of the background models used in correction procedures based on the limited amount of experimental data. Probably modifying the Galileo model via injecting NeQuick2 as a background model would provide even better results, but this requires additional studies that are beyond the scope of this current paper.

BDGIM uses the most sophisticated methodology to use broadcast coefficients compared with NeQuickG. One of the differences is that BDGIM uses a background model and disturbance to it, while NeQuickG changes whole the model based on the broadcast coefficients. Our comparison indirectly shows that the first approach could be more effective than the second one.

Finally, TEC updating procedure can improve the quality of ionospheric models (including IRI and NeQuick) [56]. Probably in the future, all the models will provide TEC updating capabilities (such as IRI-Plas) that will improve their operational effectiveness. Another way to improve ionospheric modeling is through machine learning techniques, whose usage is continuously increasing for global ionosphere modeling [37]. Such models now overperform classical models (such as IRI) [57]. These two ways seem to be the ionospheric modeling future.

## 5. Conclusions

We studied the quality of nine ionospheric models (four operational and five empirical models) in TEC and positioning domains during 2014–2020 (the full data set covered 2000–2020). Due to noises and other factors, the positioning quality involving different models (and broadcast ephemeris) does not differ much for models. We arranged (from the best) the models in the positioning domain as GIM IGSG, BDGIM, NeQuick2, GEMTEC, NeQuickG, and IRI-2016, Klobuchar, IRI-2012, IRI-Plas, GLONASS; in TEC domain as GEMTEC, BDGIM, NeQuick2, IRI-2012, NeQuickG, IRI-2016, Klobuchar, GLONASS, and IRI-Plas. 

While TEC and positioning domains differ, new-generation operational models (BDGIM and NeQuickG) overperform or at least at the same level as classical empirical models. Obtained results could show a way in which the model can be improved.

## Figures and Tables

**Table 1:** Input parameters for the ionospheric models.

Model	Input Parameters	References
Klobuchar	Broadcast coefficients	[15,16]
NeQuickG	Broadcast coefficients, CCIR	[17]
BDGIM	Broadcast coefficients	[18,19]
GLONASS ^1^	*F10.7*, *Ap*	[20]
IRI-2016	IG12, URSI, topside—IRI-corr	[9]
IRI-2012	IG12, URSI, topside—IRI-corr	[21]
IRI-Plas	IG12, URSI	[22]
NeQuick2	*F10.7*, CCIR	[10]
GEMTEC	*F10.7*	[23]

^1^ We do not have broadcast coefficients, so we used indices *F10.7*, *Ap*, rather than broadcast parameters.

**Table 2:** Mean 3D error of single-frequency positioning involving different models for ionosphere correction and mean errors of TEC. The data when all the models appeared (mostly 2015–2020).

Ionosphere Correction	σ_3D_, m	<Δ*I*>, TECU	<Δ*I*/*I*>, %	<|Δ*I*|>, TECU	<|Δ*I*|/*I*>, %
GIM IGSG	1.838	-	-	-	-
BDGIM	1.995	−0.67	17.81	2.89	44.00
NeQuick2	2.032	−1.22	−9.20	3.00	30.00
GEMTEC	2.042	−0.28	4.79	2.36	26.90
IRI-2016	2.042	−1.77	−17.06	3.15	30.48
NeQuickG	2.044	−1.53	−8.95	3.49	37.31
Klobuchar	2.082	1.24	75.98	4.84	90.81
IRI-2012	2.083	−1.47	−15.16	3.15	30.35
IRI-Plas	2.168	3.06	39.27	4.24	47.20
GLONASS	2.185	−1.91	−9.20	4.81	52.32
No correction	2.356	-	-	-	-

**Table 3:** TEC error limits for different data sets with the smallest error.

Ionosphere Correction	σ_3D_, m ^1^	25%, <TECU	50%, <TECU	75%, <TECU	90%, <TECU	95%, <TECU
BDGIM	1.995	0.9	2.3	4.7	8.1	10.8
NeQuick2	2.032	1.0	2.6	5.3	10.0	14.5
GEMTEC	2.042	0.6	1.8	4.1	8.1	11.9
IRI-2016	2.042	1.0	2.6	5.3	10.4	15.4
NeQuickG	2.044	0.8	2.2	4.3	7.4	10.4
Klobuchar	2.082	1.9	4.2	7.5	15.2	23.1
IRI-2012	2.083	1.0	2.5	5.1	9.8	14.4
IRI-Plas	2.168	1.0	2.7	5.9	11.3	15.7
GLONASS	2.185	1.9	4.4	8.7	15.0	20.5

^1^ Column “σ3D, m” just duplicates information from Table 2.

## Data Availability

Used data are publicly available. GNSS Rinex data and GIM can be accessed via EOSDIS Earthdata (https://urs.earthdata.nasa.gov/ (accessed on 1 January 2022)). *F10.7* data is available at https://spaceweather.gc.ca/forecast-prevision/solar-solaire/solarflux/sx-5-en.php (accessed on 1 January 2022); *Kp* and *Ap* is available at ftp://ftp.gfz-potsdam.de/pub/home/obs/kp-ap/wdc/ (accessed on 1 January 2022).
